# Effects of Hydrogen Dissociation During Gas Flooding on Formation of Metal Hydride Cluster Ions in Secondary Ion Mass Spectrometry

**DOI:** 10.3390/nano14211687

**Published:** 2024-10-22

**Authors:** Jernej Ekar, Sabina Markelj, Miran Mozetič, Rok Zaplotnik, Janez Kovač

**Affiliations:** 1 Jožef Stefan Institute, Jamova Cesta 39, 1000 Ljubljana, Sloveniamiran.mozetic@ijs.si (M.M.); rok.zaplotnik@ijs.si (R.Z.); 2 Jožef Stefan International Postgraduate School, Jamova Cesta 39, 1000 Ljubljana, Slovenia; 3 Elettra Sincrotrone Trieste, Strada Statale 14, 34149 Basovizza, Trieste, Italy

**Keywords:** hydrogen atmosphere, molecule dissociation, gas flooding, cluster ions, secondary ion mass spectrometry

## Abstract

The application of hydrogen flooding was recently shown to be a simple and effective approach for improved layer differentiation and interface determination during secondary ion mass spectrometry (SIMS) depth profiling of thin films, as well as an approach with potential in the field of quantitative SIMS analyses. To study the effects of hydrogen further, flooding of H_2_ molecules was compared to reactions with atomic H on samples of pure metals and their alloys. H_2_ was introduced into the analytical chamber via a capillary, which was heated to approximately 2200 K to achieve dissociation. Dissociation of H_2_ up to 30% resulted in a significant increase in the intensity of the metal hydride cluster secondary ions originating from the metallic samples. Comparison of the time scales of possible processes provided insight into the mechanism of hydride cluster secondary ion formation. Cluster ions presumably form during the recombination of the atoms and molecules from the sample and atoms and molecules adsorbed from the gas. This process occurs on the surface or just above it during the sputtering process. These findings coincide with those of previous mechanistic and computational studies.

## 1. Introduction

Secondary ion mass spectrometry (SIMS) is a widely used analytical method that provides the user with information about the elemental, molecular, and isotopic composition of a sample [1]. Analytes are detected as secondary ions formed as a consequence of bombardment of the surface with the primary ions [2]. This method is primarily surface-sensitive (to the topmost few nm) and offers measurements of mass spectra and mapping of the chemical composition of an analyte on the surface (imaging) [1,3]. If the ion current density of the primary ions is high enough, depth profiling via the application of one or two ion beams (dual-beam depth profiling) can be performed [4]. By combining 2D imaging and depth profiling, the generation of 3D representations becomes possible [3].

SIMS also has its limitations, with one of the more complex ones being the matrix effect, which can be described as the dependence of the ionization yield of the sputtered material on the substrate composition [5,6,7]. The intensity of the secondary ion current (*I*_m_) is a function of the current of the primary ions (*I*_p_), the sputter yield (*Y*_m_), the ionization probability (*α*^+/−^), the concentration of the compound analyzed (*θ*_m_), and the transmission of the analytical system (*η*). Their relation is described by Equation (1) [1].

*I*_m_ = *I*_p_*Y*_m_*α*^+/−^*θ*_m_*η* (1)

The ionization probability is significantly influenced by the effect of the electronic properties of the substrate and changes in these, as a consequence of the matrix effect, even for a few orders of magnitude [5]. The matrix effect notably affects the detection limits and almost entirely prevents quantification of the measured spectra [6]. Different approaches, such as the relative sensitivity factor (RSF) method incorporating internal standards [8,9,10] and MCs^+^ and MCs_2_^+^ analyses (M being the metal of interest) combined with reactive Cs^+^ sputtering [11,12], have been developed for at least semi-quantitative analysis. Other SIMS variations target improvement of the ionization yield and reductions in the matrix effect. These include laser [13,14,15] and electron beam [16,17,18] postionization of sputtered neutrals (secondary neutral mass spectrometry (SNMS)); metal-assisted SIMS, utilizing deposition of very thin metallic layers onto the surface of the sample [19,20,21]; matrix-enhanced SIMS, improving the ionization with an overlayer of MALDI (matrix-assisted laser desorption/ionization) matrices or ionic liquids [22,23,24]; dynamic reactive ionization (DRI), combining Ar clusters, HCl, and H_2_O molecules [25,26]; and reactive gas flooding with gases such as O_2_ and XeF_2_ [27,28,29].

H_2_ and O_2_ atmospheres were recently proven by our group to improve the capabilities of SIMS quantification significantly by reducing the matrix effect [30]. The novel H_2_ flooding approach substantially improves the capabilities of SIMS concerning depth profiling of metals, metal oxides, and alloys by providing unambiguous identification of different layers, which is otherwise impossible as a consequence of the matrix effect [31]. The presence of H_2_ during depth profiling also reduced ion-sputtering-induced surface roughening [32]. Differentiation between layers of metals and their oxides, interface clarity, and depth resolution are of high importance during research om corrosion processes [33,34,35], analysis of thin films and multilayered samples [36,37,38], characterization of nanomaterials and nanoparticles [39,40], evaluation of protective surface (oxide) layers formed during metal and alloy treatments [41,42], and the study of catalytic processes [43].

To improve the positive effects of the presence of hydrogen during SIMS analyses further and to gain insight into the mechanism of cluster secondary ion formation, we studied the influence of atomic hydrogen on alloys composed of different metals. H radicals (neutral hydrogen atoms in the ground electronic state) are more reactive than H_2_ molecules, so they are often used to enhance the intensity of surface reactions [44,45,46,47]. Hydrogen dissolves extensively in some of the metallic materials that can be used as solid storage materials [48,49], and the effects of atomic hydrogen are also of interest in terms of these properties.

During the SIMS measurements, higher intensities of the metal hydride cluster secondary ions at the same pressure of gas were observed during the operation of the hydrogen atom beam source due to the enhanced adsorption of H atoms compared to that of H_2_ molecules. The increase in the intensity of different hydride secondary ions was found to correlate with the number of H atoms/ions constituting these secondary ions. The highest increase in intensity was observed for ions composed of two or more H atoms/ions. The increase in the intensity of hydride ions also correlated with an increase in the heating power of the atom beam source, leading to a higher degree of H_2_ dissociation. The mechanism of cluster secondary ion formation upon treatment with a mixture of atomic and molecular hydrogen was discussed and evaluated as well. Through descriptive explanations and mathematically derived conclusions, it was confirmed that the formation of cluster secondary ions occurs after the adsorption of gaseous species (monolayer formation) and during the sputtering process on the surface or immediately after the particles are sputtered away just above the surface.

## 2. Materials and Methods

### 2.1. Preparation of the Samples

We used two different alloys with a homogenous distribution of elements. The composition in atomic percentages was 50% Ni and 50% Ti for the NiTi alloy and 65% Fe, 19% Cr, 12% Ni, 1.5% Mn, 1.5% Mo, and 1% Si for the stainless steel sample. The NiTi sample was characterized by the producer Alfa Aesar (Thermo Fisher, Haverhill, MA, USA), while the composition of the stainless steel sample was determined using energy-dispersive X-ray spectroscopy (EDXS) [30]. Both samples had their surfaces sufficiently polished during the process of their formation (a NiTi sheet and a stainless steel cube) to enable successful SIMS analysis. The alloys were also covered with a thin layer of native oxide, which was removed using 1 keV Cs^+^ ion sputtering prior to studying the effects of hydrogen dissociation.

### 2.2. Secondary Ion Mass Spectrometry Measurements

The ToF-SIMS analyses were performed using the TOF.SIMS 5 instrument produced by IONTOF, Münster, Germany. A Bi^+^ primary ion beam with a lateral resolution of approximately 5 µm was used for the analysis. The energy of the Bi^+^ ions was 30 keV, and the current was between 1.1 and 1.5 pA. The ion beam was pulsed with a pulse length of 7 ns, providing a mass resolution *m*/Δ*m* between 4000 and 15,000 depending on the type of secondary ion detected.

All the analyses were performed during dual-beam depth profiling, with Cs^+^ ions used as in the sputtering ion beam. Their energy was 1 keV, and their current was between 60 and 69 nA. Sputtering with the Cs^+^ ions was performed over a 400 µm × 400 µm area, while analysis with the Bi^+^ ions was performed over a 50 µm × 50 µm area located in the center of the depth profiling crater.

The H_2_ used for the gas flooding had a purity of 99.9999%. It was introduced into the analysis chamber via the hydrogen atom beam source. The pressure of the hydrogen in the analytical chamber during the measurements was 7 × 10^−7^ mbar, and the pressure before the capillary of the atom beam source (the driving pressure) was approximately 0.1 mbar. The pressure before hydrogen flooding was always approximately 1 × 10^−8^ mbar. These pressures were determined using cold cathode gauges.

### 2.3. Principles of the Hydrogen Atom Beam Source’s Operation

In these experiments, we used a commercial hydrogen atom beam source (HABS) from MBE Komponenten GmbH, Weil der Stadt, Germany (https://www.mbe-komponenten.de/gas-sources/habs/, accessed on 19 October 2024) [50]. This HABS is a thermal gas cracker that produces an ion-free hydrogen gas beam. The atomic hydrogen is generated in a hot tungsten capillary and heated by the thermal radiation from a surrounding tungsten filament up to 2200 K. The tungsten filament around the tungsten tube is resistively heated to heat the tube by thermal radiation. The heat loss is minimized by a thermal shield made of Ta. The shield is then surrounded by water-cooled copper housing. The temperature of the source is measured using a free-standing thermocouple (TC) mounted inside the thermal shield. The capillary has a temperature approximately 100–200 K higher than that measured by the TC. The W capillary, of a 1 mm inner diameter and a 10 mm length, is the only hot part of the HABS with direct contact with the hydrogen gas. The formation of a narrow-shaped atomic hydrogen beam results from the long heated area of the W tube. The tube’s inner diameter allows a gas flux of up to 0.5–1.0 standard cubic centimeters per minute (sccm). The gas flow within the W tube forms a narrow angled gas jet with a FWHM (full width at half maximum) of about 15–30°, dependent on the flow rate. At low rates, the gas beam is more focused. A basic description of the source, with some results on its characterization, is published in other literature works [50,51,52].

The hydrogen atom beam source is equipped with a tantalum recombinator that can be placed in front of the atom beam orifice, 15 mm away, without disturbing the flow. If the recombinator is opened, the H atoms are directed at the sample. If the recombinator is closed, the H atoms cannot hit the sample directly but experience a few collisions with the recombinator and the metallic walls of the analysis chamber. The H atoms are likely to stick onto metallic surfaces and recombine if the fluence of the atoms is large enough [53,54]. Therefore, the recombinator suppresses the flux of H atoms onto a sample’s surface significantly. On the other hand, the molecules that drift from the capillary are not affected much by the recombinator, except that they are thermalized during elastic collisions with surfaces, so their temperature should be lower if the recombinator is placed closer to the exhaust of the capillary.

When the capillary is not heated, the molecules experience free adiabatic expansion from the capillary to the analysis chamber, so they are close to room temperature. If the capillary is heated and the recombinator is absent, they experience relatively high temperatures. If the capillary is heated and the recombinator is positioned in between the exhaust and the sample, the temperature of hydrogen molecules is moderate. However, it was not possible to measure the gas temperature close to the sample during these experiments.

## 3. Results

### 3.1. Comparison of the Effects of Hot H_2_ Molecules and H Atoms on the SIMS Signals

**Figure 1:**
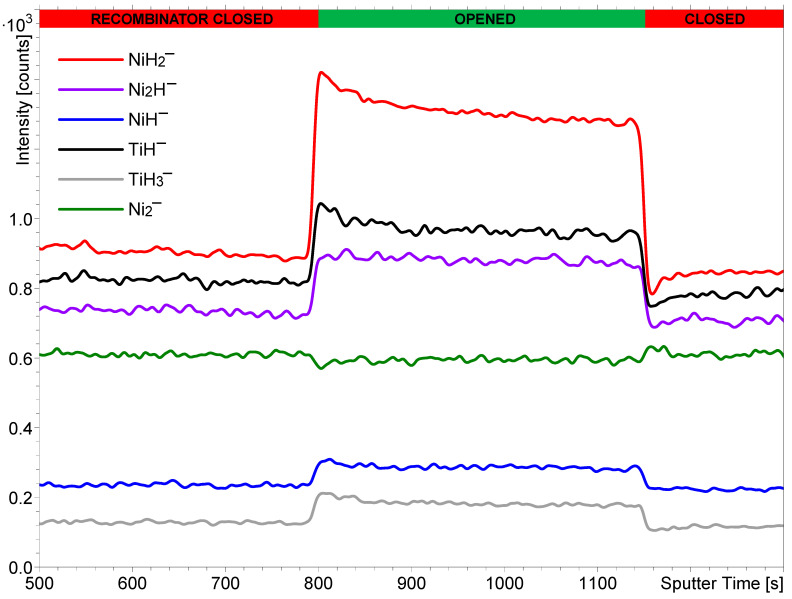
The intensity of different secondary ions in the SIMS depth profile from the NiTi sample as a function of opening or closing the recombinator in front of the hydrogen atom beam source. The profile was measured at a hydrogen pressure of 7 × 10^−7^ mbar and a hydrogen atom beam source power of 200 W. The sputter time on the x-axis corresponds to the measurement performed in the SIMS depth profiling mode, i.e., when using both Bi^+^ and Cs^+^ beams. The intensities of some of the secondary ions were multiplied by different factors to reduce the width of the y-axis.

In the first step, the effects of the heated H_2_ molecules and the H atoms were compared. Their influence on the SIMS signals was controlled via opening and closing of the recombinator in front of the hydrogen atom beam source. The effects of hydrogen dissociation were evaluated for the NiTi sample. As seen in Figure 1, the opening of the recombinator at 800 s caused an increase in intensity of all metal hydride secondary ions. Closing the recombinator at 1150 s resulted in a decrease in their intensities. The relative change in intensity was the highest for secondary ions that contain two or three hydrogen atoms/ions (NiH_2_^−^ and TiH_3_^−^), and it was approximately 50%. In the case of secondary ions with only one H atom/ion (Ni_2_H^−^, NiH^−^, and TiH^−^), this change was only between 20% and 25%. Such observations correspond to the fact that the increased reactivity of the H atoms in comparison to the H_2_ molecules and consequently their faster adsorption onto the sample surface will most significantly influence the intensity of secondary ions containing the highest number of H atoms/ions. The high density of hydrogen atoms on the surface is most important for the recombination of metal atoms with numerous H atoms. Without the presence of H_2_, the signal intensity of the metal hydride secondary ions is close to zero, as already shown previously in pressure-dependence experiments [30]. The intensity of the Ni_2_^−^ signal shows no observable change as a consequence of opening or closing the recombinator, so it can be concluded that its formation is not influenced by the increased reactivity of the H atoms. Careful monitoring of the green curve in Figure 1 might lead to the conclusion that the Ni_2_^−^ signal is lowered when the recombinator is opened and recovers to its initial value when the recombinator is closed, but this difference is marginal, so we cannot be conclusive. The main reason for the constant Ni_2_^−^ signal is the fact that it is composed of Ni only, and the higher concentration of H atoms on the surface has no direct influence on its formation. However, the adsorption of hydrogen can cause slight changes in the work function, promoting the formation of secondary ions of a specific polarity. Furthermore, as the intensities of the nickel hydride secondary ions increase, more Ni is used for their formation, and less of it remains available to be sputtered in the form of Ni*_n_*^−^, where *n* is a positive integer. These could also be reasons for the small and inconclusive changes in the intensity of Ni_2_^−^.

**Figure 2:**
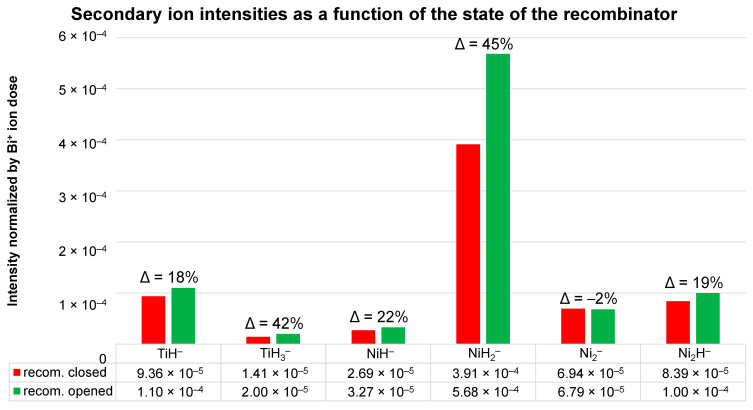
The normalized intensities of the TiH^−^, TiH_3_^−^, NiH^−^, NiH_2_^−^, Ni_2_^−^, and Ni_2_H^−^ ions integrated over the sputter times between 600 and 750 s (closed recombinator) and between 900 and 1050 s (opened recombinator) from the depth profile in Figure 1. Secondary ions were normalized by the total dose of primary Bi^+^ ions.

The normalized intensities of the TiH^−^, TiH_3_^−^, NiH^−^, NiH_2_^−^, Ni_2_^−^, and Ni_2_H^−^ ions are also plotted in Figure 2. Their intensities were integrated over the sputter time between 600 and 750 s (with the recombinator closed) and the sputter time between 900 and 1050 s (with the recombinator opened) at a hydrogen pressure of 7 × 10^−7^ mbar and a hydrogen atom beam source power of 200 W. The changes in intensity in different secondary ions depending on the state of the recombinator clearly indicate the observation already noted of the larger differences in intensity present for hydride cluster ions containing two or three hydrogen atoms/ions.

Here, it is worth mentioning that the analyses of both the NiTi and stainless steel samples were performed during SIMS depth profiling using Bi^+^ and Cs^+^ ion beams. This approach was necessary to remove the surface oxide layer. This is why the x-axis in Figure 1 does not originate at the sputtering time of 0 s. It is also well known that depth profiling with Cs^+^ ions results in the implantation of cesium onto a sample’s surface. The presence of cesium causes a reduction in the work function and a consequent enhancement of the ionization of negative secondary ions [28,55]. Since metal hydrides are ionized more efficiently in negative polarity than in positive polarity, Cs^+^ sputtering increases the intensity of the MH*_n_*^−^ signals, with M being the metal, thus increasing the signal-to-noise ratio.

### 3.2. Effect of the Hydrogen Atom Beam Source’s Power on the Secondary Ion Intensity

**Figure 3:**
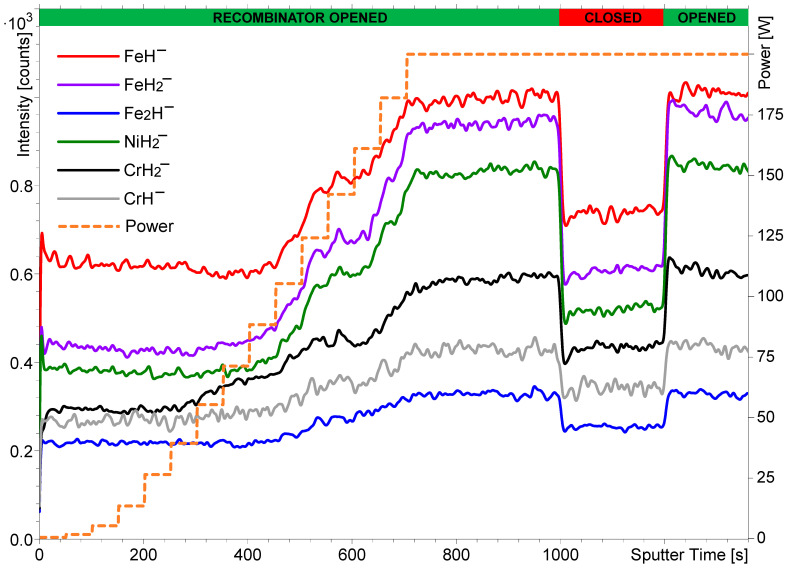
The changes in intensity of different hydride secondary ions from the stainless steel sample as a function of increasing the hydrogen atom beam source’s power from 0 to 200 W and opening or closing the recombinator in front of the hydrogen atom beam source. The powers and the state of the recombinator are noted on the upper x-axis. The profile was measured at a hydrogen pressure of 7 × 10^−7^ mbar. The intensities of some of the secondary ions were multiplied by different factors to reduce the width of the y-axis.

The next set of experiments was performed using the stainless steel sample. The intensity of the metal hydride cluster secondary ions formed during H_2_ flooding depends on the pressure of the H_2_ in the analytical chamber [30]. A similar dependence on the proportion of atomic H compared to molecular H_2_ was expected. Since the ratio of molecular to atomic hydrogen depends on the working power of the hydrogen atom beam source, the intensity of the metal hydride signals was measured against the power at a constant hydrogen pressure of 7 × 10^−7^ mbar. The hydrogen atom beam source’s power was gradually increased from 0 to 200 W, which means the temperature of the capillary was gradually increased. H_2_ starts to dissociate into H atoms when the temperature is above 1500 K. At a maximum power of 200 W, the fraction of H_2_ dissociation expected is approximately 30% [50]. Figure 3 shows the intensities of different hydride cluster secondary ions as a function of the hydrogen atom beam source’s power and the state of the recombinator. The source power versus the sputtering time is also presented in Figure 3 and corresponds to the lower temperature limit required for H_2_ dissociation accordingly.

An increase in the intensity of the metal hydride ions can indeed be observed during the increase in the atom beam source’s power. The most pronounced change in intensity is present between 100 and 200 W, which corresponds to the sputtering time between 450 and 700 s. Below 100 W, when the temperature is below or around 1000 K, these changes are minimal. The effect of closing (1000 s) and opening (1200 s) the hydrogen atom beam source recombinator again was tested, and these results were qualitatively the same as observed when using the NiTi sample (Figure 1). The same trend was also observed regarding the relative change in intensity depending on the number of H atoms/ions constituting the secondary ions. However, a slight difference in the intensity of the secondary ions shown in Figure 3 can be observed if the power is 0 W and the recombinator is opened (the sputter time between 0 and 50 s) and if the power is 200 W and the recombinator is closed (the sputter time between 1000 and 1200 s). The slightly higher intensity at 200 W can be explained by the incomplete recombination of the H atoms despite the closure of the recombinator and/or by the higher kinetic energy of the heated H_2_ molecules as a result of the hydrogen atom beam source heating them at approximately 2200 K. Incomplete recombination of H atoms on the surface of solid materials (with a recombination coefficient < 1) has been reported by numerous authors on numerous materials [56,57,58,59]. The coefficient for other atoms like O has never been reported to be above 0.1 and is typically in the order of 0.1 for many metals and alloys [60].

**Figure 4:**
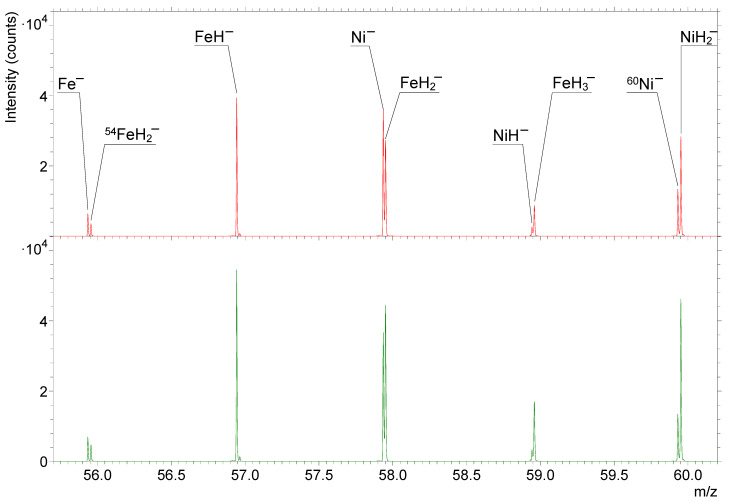
The intensities of the Fe and Ni signals from the stainless steel sample in the *m*/*z* range from 55.7 to 60.2. The upper red spectrum was measured between 1025 and 1175 s in the depth profile from Figure 3 (with the recombinator closed). The lower green spectrum was measured between 1225 and 1375 s in that same depth profile (with the recombinator opened). The hydrogen pressure was 7 × 10^−7^ mbar, and the power of the hydrogen atom beam source was 200 W.

In Figure 4, mass spectra in the *m*/*z* range between 55.7 and 60.2 are presented. The Fe and Ni signals from the stainless steel sample analyzed at the hydrogen pressure of 7 × 10^−7^ mbar and atom beam source power of 200 W are shown. The upper, red spectrum was measured when the recombinator was closed, and the lower, green spectrum when the recombinator was opened. It can be clearly seen that the opening of the recombinator causes an increase in the intensity of the larger-exact-mass signal for each nominal mass pair (Fe^−^ and ^54^FeH_2_^−^, Ni^−^ and FeH_2_^−^, NiH^−^ and FeH_3_^−^, and ^60^Ni^−^ and NiH_2_^−^). The signals with the larger mass (the ones on the right) always have the larger number of hydrogen atoms/ions, thus proving that the highest intensity increase is observed for the secondary ions with the larger number of hydrogen atoms/ions.

## 4. Discussion

### 4.1. Adsorption Affinity of H_2_ and H

Figure 1, Figure 2, Figure 3 and Figure 4 clearly show that the dissociation of hydrogen molecules causes an increase in the intensity of the metal hydride secondary ions. The reason for this is most probably the higher adsorption rate of atomic H due to its higher reactivity. Figure 3 also indicates that heating the H_2_, despite it remaining in the molecular form, will also have a similar effect but to a much smaller extent. By increasing the kinetic energy of molecules, their reactivity becomes higher, and the probability of successful adsorption increases. However, the effect of the heating applied during our experiments affected the probability of metal hydride secondary ion formation to a significantly lesser extent than the dissociation and formation of H radicals. As already described, hydrogen dissociation influences metal hydride secondary ion formation to different extents depending on the number of H atoms/ions constituting the metal hydride ions in question. The requirement for a higher surface hydrogen density for the formation of secondary ions composed of numerous H atoms/ions (MH*_n_*^−^, *n* > 1) is the reason for their relatively higher change in intensity in comparison to that of monohydride ions (MH^−^).

### 4.2. Mechanism of Metal Hydride Secondary Ion Formation

Cluster secondary ions can theoretically form on the surface or just above it during the sputtering process or in the plume of sputtered particles in the vacuum after the completion of the sputtering process due to collisions with gaseous atoms and molecules. Previous studies have indicated that the recombination process indeed happens on the surface [61,62,63] or just above it [61,64,65], and similar conclusions can be obtained from the results of our hydrogen flooding experiments. Firstly, the formation of high-intensity secondary ions such as TiH_3_^−^ in the vacuum would require a high rate of occurrence of successful three-particle collision reactions, which is highly unlikely. In the case of atomic H, a three-particle reaction would be partially required for the formation of dihydride ions as well, although it would not be completely necessary due to the incomplete dissociation of hydrogen. If the in-vacuum formation of cluster secondary ions would indeed be their main formation pathway, then according to particle collision reactions, the dissociation of hydrogen would most significantly increase the intensity of monohydride secondary ions. This is not the case since H_2_ dissociation results in the most pronounced increase in intensity of hydride secondary ions composed of numerous H atoms/ions. Already, based on this, we can conclude that the formation of hydride cluster secondary ions originating from the metallic surfaces does not primarily occur in the vacuum.

The next argument against the in-vacuum formation of hydride cluster ions is the time scale of the collisions and the time available for successful reactions to occur. The latter corresponds to the time frame between the primary ion pulse and the end of the extraction into the analyzer, which is a few µs at most. The average time required for collisions to occur can be calculated via the kinetic theory of gases. The root mean square (RMS) speed of the gas can be calculated via Equation (2) [66]:

(2) $$v_{\mathrm{RMS}}=\sqrt{\frac{3\mathrm{RT}}{M}}$$

where *v*_RMS_ is the RMS speed of the gas particle, *R* is the molar gas constant of 8.31 J/molK, *T* is the temperature in K, and *M* is the molar mass. The thermal temperature of the hydrogen molecules exiting the hydrogen atom beam source depends on the heating power and can be up to 2200 K. When H atoms or H_2_ molecules hit the recombinator, they lose some of their kinetic energy, and they cool down. The same thing happens when these molecules hit the chamber walls. Therefore, it is practically impossible to determine the temperature of the molecules when they reach the sample. Nevertheless, if we estimate a gas temperature of approximately 1000 K, the speed of the H_2_ molecules can be calculated as approximately 3500 m/s and the speed of H atoms as 5000 m/s. According to Equation (3),

(3) $$l=\frac{k_{B}T}{\sqrt{2}\pi d_{1}d_{2}p},$$

and the mean free path of the gaseous particle can be calculated as well [66]. Here, *l* denotes the mean free path, *k*_B_ the Boltzmann constant of 1.38 × 10^−23^ J/K, *d* the diameter of the colliding particles, and *p* the pressure in Pa, which was 7 × 10^−5^ Pa. Since collisions of H_2_ molecules or H atoms with metal atoms are of interest, *d*_1_ and *d*_2_ should correspond to the diameters of the metal atoms and the H_2_ molecules or H atoms. There are also possible deviations in these diameters due to the potential ionization of the atoms or molecules, but for the purpose of this discussion, these will be disregarded. Fe, with an atomic diameter of 252 pm [67], can be chosen as a common example of a metal atom. The kinetic and van der Waals diameters of a H_2_ molecule and a H atom are 289 [68] and 220 pm [69], respectively. Therefore, mean free paths of 610 m for the H_2_ molecules and 800 m for the H atoms can be calculated. The average times required for metal–H_2_/H collisions (*t*) can be determined via Equation (4):

(4) $$t=\frac{l}{v}.$$

The values of 170 ms (H_2_) and 160 ms (H) are more than four orders of magnitude larger than the time frame during which the collision reactions can occur. This is another indicator of the low probability that in-vacuum collision reactions represent a significant pathway for metal hydride cluster ion formation.

The formation of hydride cluster ions on the surface of the sputtered samples will consequently be considered in the following. Comparison of the monolayer formation via gas adsorption and its removal via sputtering indicates a high probability of this mechanism being the most important one. Continuing the analysis considering iron, its numerical density of 8.5 × 10^28^ atoms/m^3^ can be calculated via Equation (5):

(5) $$\rho_{N}=\frac{\rho_{m}N_{A}}{M}$$

where *ρ*_N_ represents the numerical density, *ρ*_m_ the mass density, and *N*_A_ the Avogadro constant of 6.02 × 10^23^ mol^−1^. The number of Fe atoms per meter can be obtained from the value of *ρ*_N_ and is 4.4 × 10^9^ atoms/m. This value can be transformed further into the size of the atoms, although due to the arrangement of the atoms in the crystal structure of the metal, it is closer to the thickness of the monolayer, which is 230 pm. Due to the tightly packed atoms in the solid Fe, this value corresponds appropriately to the slightly larger diameter of Fe atoms of 252 pm [67]. The sputter rate of iron in the H_2_ atmosphere with 1 keV Cs^+^ ions and under the same analysis conditions was measured as 84 pm/s, which can be translated into 0.37 monolayer/s. Therefore, approximately 2.7 s are needed to sputter one monolayer of Fe with adsorbed hydrogen.

The time needed for the complete formation of a hydrogen monolayer can be determined as well. If a sticking coefficient of 1 (each atom that hits the surface is also adsorbed) and the adsorption of one gas particle per atom on the surface are assumed, then Equation (6),

(6) $$\tau =\frac{4Dk_{B}T}{pv_{\mathrm{RMS}}},$$

can be applied. *τ* is the time needed for the monolayer formation, and *D* is the dose of gas particles that, according to our assumption, equals the surface density of Fe atoms (1.9 × 10^19^ atoms/m^2^). At a temperature of 1000 K and a pressure of 7 × 10^−5^ Pa, *τ* equals 4.3 s for the H_2_ molecules and 3.0 s for the exclusively H atoms. Equation (6) is derived from Equation (7) [70],

(7) D=jτ,

where *j* represents the current of the gas molecules and is defined via Equation (8) [70] as

(8) $$j=\frac{\rho_{G}v_{\mathrm{RMS}}}{4}.$$

*v*_RMS_ is defined by Equation (2), and *ρ*_G_ is the density of the gas in particles/m^3^ defined by Equation (9):

(9) $$\rho_{G}=\frac{p}{k_{B}T}.$$

In the case of the recombinator in front of the hydrogen atom beam source being opened, Equation (6) does not describe the process of adsorption completely since gas is introduced via the point source. However, considering the distance from the source to the sample and the driving pressure of the atom beam source in relation to previously measured gas currents [71], it can be concluded that the gas current in this system is even lower if the point source is considered instead of a random distribution. The latter gives a value of *j* in the range of 10^18^ m^−2^s^−1^, while calculation via approximation of the point source gives a value of *j* in the range of 10^17^ m^−2^s^−1^. A lower current of gas molecules *j* results in an even longer monolayer formation time *τ*.

Furthermore, the assumption of dose *D* being equal to the surface density of the metal ions is not necessarily correct. A comparison with Equation (10),

(10) $$\tau =\frac{3.2\;\times \;10^{\text{−}4}\;\mathrm{Pa}\;\times \;s}{p}$$

which represents an approximation of the time needed for gas molecules to form one monolayer, that is, the monolayer formation time [72], is consequently sensible. The approximation in Equation (10) also assumes a sticking coefficient of 1. At a pressure of 7 × 10^−5^ Pa, *τ* as calculated via Equation (10) equals 4.6 s for both H_2_ and H. The monolayer formation time in this case is slightly longer than that when assuming equal values for the gas dose and the surface density of metal ions. It is consequently possible to conclude that slightly more than one H atom is adsorbed into each Fe atom. Such a conclusion corresponds to the notable size difference between Fe and H atoms. Furthermore, although hydrogen is a relatively reactive gas and freshly sputtered surfaces are proven to adsorb gaseous species at a high rate [73], the sticking coefficient of 1, especially in the case of H_2_, is too high. Since the dissociation ratio of H_2_ molecules reaches approximately 30%, a sticking coefficient below 1 can be expected during SIMS analyses combined with the use of the hydrogen atom beam source as well. The adsorption of H_2_ and H onto the surface, as well as their dissociation and recombination, is also affected by the roughness and polycrystallinity of the surfaces analyzed. However, all the samples were, regardless of the state of the atom beam source, analyzed under the same conditions, so these effects were the same for molecular and atomic hydrogen.

The consequence of a lower sticking coefficient is an even longer monolayer formation time. When accounting for all of the above effects, it is possible to conclude with a relatively high degree of certainty that the monolayer formation time under the given conditions, especially concerning H_2_ molecules, is notably longer (almost twofold) than the time needed for the removal of one monolayer of Fe with adsorbed hydrogen, which accounts for 2.7 s. Dissociation accelerates the monolayer formation through the adsorption of more reactive H atoms with a higher sticking coefficient, therefore creating a larger portion of the monolayer before the latter is sputtered away. Since molecule dissociation also causes an increase in the intensity of the metal hydride secondary ions, it can be concluded that a higher degree of hydrogen adsorption and a higher percentage of hydrogen monolayer formation increase the formation rate of hydride secondary ions. This correlation proves the importance of the adsorbed species and indicates that cluster secondary ions are preferentially formed on the surface or just above it during the sputtering process, with the mechanism being the recombination of sample particles with pre-adsorbed atoms and molecules.

## 5. Conclusions

A significant increase in the intensity of metal hydride secondary ions was observed after the dissociation of hydrogen molecules, with much lesser effects caused by heated but undissociated H_2_. The increased formation rate of the metal hydride secondary ions is a consequence of the higher reactivity of H atoms in comparison to H_2_ molecules. H atoms are more easily adsorbed onto the freshly sputtered surface, with the consequence being faster hydrogen monolayer formation. The correlation of the monolayer formation and the sputter rate with the intensity of the hydride secondary ions indicates that cluster secondary ions are formed by the recombination of atoms and molecules from the sample with atoms and molecules adsorbed from the gas on the surface or just above it during the sputtering process. The portion of cluster secondary ions formed in the vacuum after sputtering is minimal, if present at all. However, these mechanistic explanations would benefit from additional studies to evaluate them further. The most straightforward approach, planned for future publication, is a comparison of the effects of different sputter rates, controlled via the sputtering parameters, and different monolayer formation times, controlled via the gas pressure.

## Data Availability

The additional data in the form of the original SIMS spectra and depth profiles will be made available on request. The reason for this is the format in which the measurements are encoded. If the potential user does not have the appropriate software at their disposal, we will discuss what form they want the data in with them (for example, ASCII). Furthermore, the measurements are accompanied by written notes considering the changes in the output power of the hydrogen atom beam source and the closing or opening of the recombinator at specific times. Since the hydrogen atom beam source is not an original part of the TOF.SIMS 5 instrument, such information is not recognized or recorded by the IONTOF software SurfaceLab 7 or older. We consider explaining the details of the data interpretation to a potential user to be much more effective and preventative of unwanted misunderstandings, so we have decided to opt for this approach of including our help.
